# Health Technology Assessment of Belimumab: A New Monoclonal Antibody for the Treatment of Systemic Lupus Erythematosus

**DOI:** 10.1155/2014/704207

**Published:** 2014-08-17

**Authors:** Maria Lucia Specchia, Chiara de Waure, Maria Rosaria Gualano, Andrea Doria, Giuseppe Turchetti, Lara Pippo, Francesco Di Nardo, Silvio Capizzi, Chiara Cadeddu, Flavia Kheiraoui, Luca Iaccarino, Francesca Pierotti, Ilaria Palla, Maria Assunta Veneziano, Daniela Gliubizzi, Antonella Sferrazza, Nicola Nicolotti, Rolando Porcasi, Giuseppe La Torre, Maria Luisa Di Pietro, Walter Ricciardi

**Affiliations:** ^1^Institute of Public Health, Catholic University of the Sacred Heart, 00168 Rome, Italy; ^2^Department of Public Health, University of Turin, 10126 Turin, Italy; ^3^Rheumatology Department, Padua Hospital, 35128 Padua, Italy; ^4^Institute of Management, Scuola Superiore Sant'Anna, 56127 Pisa, Italy; ^5^GSK, 37135 Verona, Italy; ^6^Department of Public Health and Infectious Diseases, Sapienza University of Rome, 00185 Rome, Italy

## Abstract

*Objective.* Systemic lupus erythematosus (SLE) is treated with anti-inflammatory and immunosuppressive drugs and off-label biologics. Belimumab is the first biologic approved after 50 years as an add-on therapy for active disease. This paper summarizes a health technology assessment performed in Italy. *Methods.* SLE epidemiology and burden were assessed using the best published international and national evidences and efficacy and safety of belimumab were synthesized using clinical data. A cost-effectiveness analysis was performed by a lifetime microsimulation model comparing belimumab to standard of care (SoC). Organizational and ethical implications were discussed. *Results.* Literature review showed that SLE affects 47 per 100,000 people for a total of 28,500 patients in Italy, 50% of whom are affected by active form of the disease despite SoC. These patients, if autoantibodies and anti-dsDNA positive with low complement, are eligible for belimumab. SLE determines work disability and a 2–5-fold increase in mortality. Belimumab with SoC may prevent 4,742 flares in three years being cost-effective with an incremental cost-effectiveness ratio of €32,859 per quality adjusted life year gained. From the organizational perspective, the development of clear and comprehensive clinical pathways is crucial. *Conclusions.* The assessment supports the use of belimumab into the SLE treatment paradigm in Italy.

## 1. Introduction

Systemic lupus erythematosus (SLE) is a chronic inflammatory autoimmune disease harming skin, joints, kidneys, lungs, nervous system, and serous membranes which mostly occurs in fertile women [1]. The feature that affects patient's long term survival is tissue damage, especially when organs such as kidneys are involved [2–4].

Recent EULAR (EUropean League Against Rheumatism) recommendations for the management of patients affected by SLE were published in 2008 [5]. In 2010, EULAR recommendations for neuropsychiatric lupus were also defined [6] while, in 2012, the American College of Rheumatology (ACR) released recommendations for the management of lupus nephritis [7].

In the last 50 years no new drugs for SLE have been approved. Therapy for SLE includes nonsteroidal anti-inflammatory drugs (NSAIDs), corticosteroids, antimalarial agents, and immunosuppressant drugs [8]. None of these treatments has a specific target; rather, their aim is the reduction of inflammation and unspecific suppression of the immune system. In the last 15 years immunosuppressant and immunomodulating drugs, which act on specific target immune cells, were added as second-line treatment. Despite the large availability of these treatments, approximately 50% of patients have persistence of active SLE or the occurrence of a relapse [9, 10] which both require modifications of therapy, most commonly with an increase of corticosteroid dosage and introduction of immunosuppressant drugs [8].

This paper summarizes the results of a health technology assessment (HTA) of belimumab, which was approved at the dosage of 10 mg/kg by the US Food and Drug Administration (FDA) on March 2011 and by the European Medicines Agency (EMA) on July 2011.

Belimumab is indicated as an add-on treatment for SLE in adults with a positive autoantibody test whose disease is still highly active (e.g., anti-dsDNA positive and low complement) despite standard treatment with the exception of patients with severe active lupus nephritis or severe active central nervous system lupus [11]. Because of the unmet therapeutic need of these patients, belimumab could be a much awaited treatment from both physicians and patients and may potentially change the therapeutic framework of SLE in Italy.

The HTA of belimumab aims to fill the need for knowledge about this treatment and to establish a good basis for its proper use. Moreover the HTA may direct the design of further research and the information or training of health professionals and/or patients. The final purpose is thus to contribute to a greater effectiveness and efficiency of the decision making process.

## 2. Methods

An HTA was performed to evaluate the value of using belimumab for treating patients affected by SLE in the Italian context.

### 2.1. Epidemiology of the Disease

Target condition and population were defined using systematic reviews of the literature. Studies published on PubMed dealing with prevalence and incidence of SLE worldwide and in Italy were examined to define the frequency of the disease. The same approach was used to address the frequency of people with disability due to SLE. In order to perform these reviews the following keywords were used: “Lupus Erythematosus, Systemic” [Mesh], LES, “Epidemiology” [Mesh], incidence, prevalence, burden, and frequency.

### 2.2. Efficacy and Safety of Belimumab

A literature search was performed in PubMed and Embase to identify randomized clinical trials (RCTs) reporting the efficacy and safety of belimumab. In order to perform these reviews the following keywords were used: Belimumab, Efficacy, Safety, “Clinical Trial.” Moreover European Public Assessment Report (EPAR) and data supplied by marketing authorization holder (GlaxoSmithKline) were reported.

### 2.3. Cost-Effectiveness Analysis

A cost-effectiveness analysis was performed from both the Italian National Health Service (NHS) and societal perspectives. In particular, a microsimulation cost-effectiveness model was developed to assess the cost-effectiveness of belimumab (10 mg/kg) + the standard of care (SoC) compared to the SoC alone. The model was developed by Pharmerit International Company on Excel software and then adapted to the Italian context.  Table 1 shows the main features of the economic model.

The long term outcomes used in the model were based on data from the BLISS trials [12] and the Johns Hopkins observational cohort study [13]. The BLISS trials informed the likelihood of response at week 24, the change in SELENA-SLEDAI (Systemic Lupus Erythematosus Disease Activity Index) score up to week 52, the likelihood of discontinuation, and the effect of SELENA-SLEDAI score on utility and treatment costs. The efficacy recorded in the trials was projected over the 10-year maximum treatment period, in accordance with NICE report [14]. The main parameters used in the base case scenario are shown in Table 5.

Cost data (€, 2011) were collected from the National Tariffs [15] and, when not available, through the international literature adjusting for currency and inflation [16–19].

As the analysis was conducted from both societal and NHS perspectives, direct and indirect costs were considered [20, 21]. In particular, direct costs were related to diagnostic tests, specialist visits, and organ damage. Regarding indirect costs, the human capital approach was followed to carry out the analysis.

**Figure 1:**
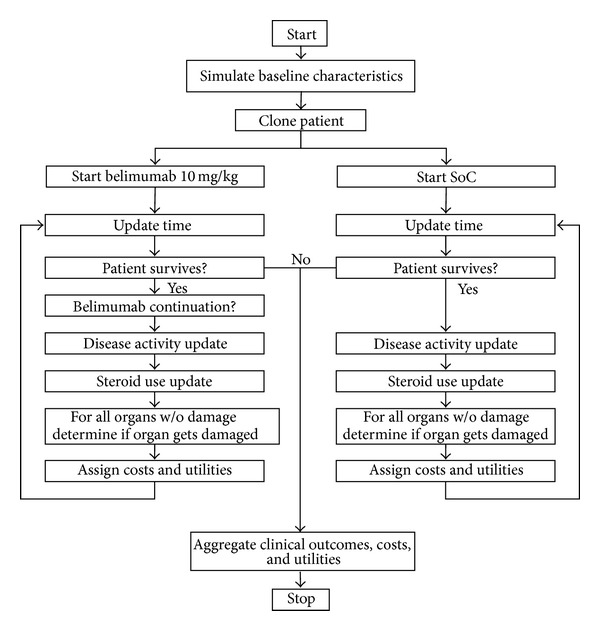
Pathways followed by patients entering the two different treatments.

Utility data, used to calculate quality adjusted life years (QALYs) related to the different health states (Figure 1), were retrieved from international literature, on the basis of the utilities elicited within the BLISS trial that considered a representative sample of UK patients [14].

The horizon of the analysis was lifetime and costs and benefits were discounted at 3% yearly. Results were reported as incremental cost-effectiveness ratio (ICER), expressed in terms of incremental cost per life year (LY) gained and QALY gained.

To assess the robustness of the base case results, univariate and probabilistic sensitivity analyses (PSA) were conducted on the following critical parameters (the distribution is reported in brackets):

- change in SELENA-SLEDAI score at week 52 (multivariate normal distribution);
- change in SELENA-SLEDAI score according to the natural history model (multivariate normal distribution);
- discontinuation rate (normal distribution);
- probability of response (gamma distribution);
- mortality and organ damage development probabilities according to the natural history model (multivariate normal distribution);
- standardized mortality rates (normal distribution);
- utility values (multivariate normal distribution);
- organ damage disutility (gamma distribution);
- costs associated with each SELENA-SLEDAI score (gamma distribution);
- organ damage costs (gamma distribution);
- indirect costs (normal distribution).

A cost-effectiveness acceptability curve (CEAC) was reported to assess how the probability of cost-effectiveness of belimumab varies according to different threshold values.

### 2.4. Organizational Aspects and Impacts

A literature review was performed to analyze health needs, healthcare priorities, and quality of life (QoL) of patients with SLE. These aspects were identified as a result of a discussion with key opinion leaders involved in SLE management. The keywords used to perform the review were “Lupus Erythematosus, Systemic” [Mesh], LES, health needs, healthcare priorities, and quality of life.

### 2.5. Ethical Evaluation

The ethical issues linked to the utilization of the product were taken into account through a framework including epistemological data, anthropologic reference, and ethical evaluation. With respect to human values, the following elements were considered: risk/benefit ratio, QoL, patient's autonomy, and social justice.

## 3. Results

### 3.1. Epidemiology of the Disease

SLE is due to both genetic and environmental factors which leads to a deregulation of the immune response. The diagnosis relies on clinical anamnesis, medical investigation, and laboratory tests which are useful to exclude different diseases. Eleven criteria were developed and provided by the ACR to make diagnosis [22] and have been recently revised and validated by the Systemic Lupus International Collaborating Clinics (SLICC) group [23]. The diagnosis is often late because of the insidious onset [24]. The disease has a remitting-relapsing pattern with the occurrence of flares, with objective increase in disease activity marked by onset or worsening of signs and symptoms [2, 3, 25]. The SLEDAI, the British Isles Lupus Assessment Group (BILAG), the Physician's Global Assessment (PGA), and the SLE Responder Index (SRI) were developed to assess disease activity.

The disease determines joint pain, which occurs in about 90% of patients [1, 26, 27]; skin rashes, which develop during the course of the disease in 85% of patients [1, 24, 26–28]; glomerulonephritis, in about 50% of patients, which may cause renal failure in 20% [28–30].

The disease is more common in non-Caucasian people, in particular Black and Hispanic [31, 32]. It affects women in 80–90% of cases with a female/male ratio ranging between 6 and 10 [31, 32]. The peak of incidence is reached between 15 and 44 years of age [31, 32].

The systematic review of the literature yielded 29 studies performed in Europe or America from 1980 on. Twenty-one studies were carried out in Europe on people belonging to Caucasian race mainly. The prevalence varied from 20 to 50 cases per 100,000 while the incidence ranged from 2 to 5 cases per 100,000 each year.

In Italy only two small studies addressed the epidemiology of SLE [33, 34]: prevalence ranged between 57.9 and 71 cases per 100,000 while incidence varied from 1.15 to 2.6 per 100,000. To calculate the population eligible to receive belimumab, data were searched in the literature, specifically, the percentage of patients with active disease and low complement levels. Chronic active disease was defined according to a SLEDAI-2K ≥ 2 (excluding the serology) in at least two out of three annual medical examinations, whereas the relapsing-remitting disease was defined as a SLEDAI-2K ≥ 2 in at least one out of three annual medical examinations. Two studies released estimates of patients responding to these criteria [9, 10] for a mean value of 50%. A direct estimate of the presence of low complement together with anti-ds DNA positivity was provided by the Systemic Lupus Erythematosus Cost of Care In Europe Study (LUCIE) [35] which yielded a value of 39.6%. Considering a mean prevalence of 0.047% [36] the population of patients affected by SLE in Italy would be approximately 28,500, whereas the population eligible to receive belimumab would be approximately 5,300.

The survival of SLE patients has improved throughout the years and it is now over 90% at 5 and 10 years [37, 38]. Daily life activities most influenced by the disease include vigorous physical activities in 83.9% of cases, housework in 79.4%, sleep in 72.9%, work activities in 70.7%, and household business in 67.8% [39]. About one-third of patients became unable to work and are obliged to retire after 3–12 years following the diagnosis.

### 3.2. Efficacy and Safety of Belimumab

The search identified one phase I study (LBSL01) [40], one randomized, double blinded phase II study controlled with placebo (LBSL02) [41], two randomized, double blinded phase III studies controlled with placebo (C1056 or BLISS-76 and C1057 or BLISS-52) [42, 43], and a combined analysis of phase III clinical trials [12].

Main results of the phase II and III trials on belimumab are reported in Table 2. In the BLISS-76 and BLISS-52 phase III pivotal trials, the study population was treated with belimumab and SoC (corticosteroids, antimalarial agents, NSAIDs, cytotoxic chemotherapy, and immunosuppressive or immunomodulatory drugs) while controls received SoC plus placebo. Primary endpoint was a reduction in the SRI at week 52 in both studies. Secondary endpoints were flares frequency, time between flares (BLISS-76 study only), and effect of the treatment on corticosteroids dosage. All endpoints underwent an intention-to-treat analysis. Two belimumab doses were studied (1 mg/kg and 10 mg/kg). In both phase III trials, the primary endpoint was achieved in a significantly greater proportion of patients treated with the 10 mg/kg dosage compared to patients treated with placebo (*P* = 0.0006 in BLISS-52 and *P* = 0.02 in BLISS-76). On the contrary, no statistically significant differences were observed between 1 mg/kg belimumab and placebo groups in the BLISS-76 study. A greater response to 10 mg/kg belimumab was also observed in the subgroup with more active disease (placebo: 31.7%; belimumab 1 mg/kg: 41.5%, *P* = 0.002; belimumab 10 mg/kg: 51.5%, *P* < 0.0001). This response maintained a statistically significant value even at week 76 only in the 10 mg/kg arm.

The combined analysis was performed collecting data from 1,684 patients enrolled in the two phase III studies. This analysis confirmed the results of the BLISS-52 and BLISS-76 trials and showed that belimumab allowed a significantly greater number of patients to reduce the prednisone dosage below 7.5 mg/die (18% in the 10 mg/kg group compared to 12% in controls, *P* < 0.05). Also, the average number of flares/year per patient was significantly lower in the 10 mg/kg belimumab group compared to controls (2.9 versus 3.5, *P* < 0.001), but no significant differences were observed in the number of severe flares/year (0.8 in the 10 mg/kg group compared to 1.0 in controls).

The safety profile of both doses of belimumab (1 mg/kg and 10 mg/kg) indicated that they were generally well tolerated. There were no significant differences between belimumab and placebo in terms of overall and serious dose-dependent adverse events. No differences were observed in events leading to discontinuation of treatment. The majority of adverse events were mild or moderate.  Table 3 shows the most common adverse events observed in phase II and III trials [41–43].

### 3.3. Cost-Effectiveness Analysis

The cost-effectiveness analysis showed that belimumab was cost-effective at the base case. In particular, from the Italian NHS perspective, ICER was equal to *€*22,990 and *€*32,859, respectively, for LY and QALY gained (Table 4). The results from the societal perspective confirmed that belimumab can be considered even more cost-effective achieving *€*20,119 for LY gained and *€*28,754 for QALY gained.

**Figure 2:**
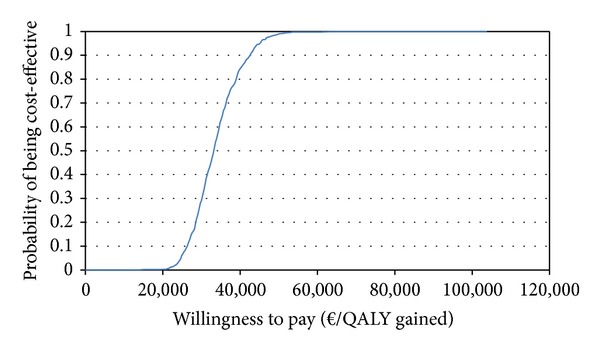
Probability of cost-effectiveness of belimumab according to the cost-effectiveness acceptability curve.

The base case results were confirmed by the PSA. The PSA showed that when the threshold/QALY was equal to *€*30,000, belimumab was 29.1% more likely to be cost-effective compared to the SoC. The CEAC showed that, when the willingness to pay/QALY was equal to *€*40,000, belimumab was 84.3% more likely to be cost-effective (Figure 2). The univariate sensitivity analysis showed that main drivers of cost-effectiveness were the treatment effect and the discontinuation rate.

In conclusion, on the basis of CEAC, it is possible to state that the introduction of belimumab within the Italian context could be recommended as it is cost-effective from both NHS and societal perspectives.

### 3.4. Organizational Aspects and Impacts

The functional status of the patients with SLE, especially during the phase of active disease, is generally compromised when compared with that of the general population. Patients also show a decrease in QoL in the sphere of both physical and emotional functions. The reduction of Health-Related Quality of Life (HR-QoL) is comparable to that of serious diseases such as acquired immunodeficiency syndrome (AIDS) or other chronic diseases such as rheumatoid arthritis, hypertension, congestive heart failure, diabetes mellitus, and myocardial infarction [44, 45].

The available studies concerning the impact of belimumab on QoL showed a significant improvement in QoL in comparison to the control group (SoC), confirming the positive effect of the drug on the different dimensions of HR-QoL [46, 47].

The diagnosis of SLE is often late, largely due to the insidious onset of the disease [24]. Patients frequently need the advice and active cooperation of several specialists (rheumatologists, internists, nephrologists, immunologists, and dermatologists) and are hampered by the low prevalence of the disease [36], the late diagnosis [24], the strong inter- and intraregional heterogeneity in accessing new therapies—especially in the case of infusion therapies—and the lack of specific clinical pathways.

According to the results of a survey carried out in Italy in 2011, SLE is treated in one out of four hospitals, and only 55% of centers treating SLE provide patients with intravenous biological drugs commonly used in the treatment of other diseases [48]. The complexity of taking charge of the patient and the use of off-label drugs represent further issues to be considered. In this context, belimumab would help to bridge the therapeutic gap for SLE, by adding value in terms of offering appropriate treatments and improvement of QoL.

### 3.5. Ethical Evaluation

On the basis of phase III clinical trials, belimumab has a favorable risk-benefit profile even though further studies are needed to address the safety profile outside clinical research studies. Available studies also showed that belimumab can improve QoL. An adequate communication process about the possible risks and benefits, way of administering, and follow-up schedule, is required to guarantee the autonomy of the patient. The involvement of general practitioners (GPs), if integrated with specialist centers, might be an appropriate solution for diagnosis and timely initiation of therapy and to facilitate the communication process. The only critical point seems to be social justice, which is threatened by economic constraints and heterogeneity in access to care within Italy.

To guarantee the correct use of belimumab, several actions are needed: the promotion of integration between primary care and specialists, in order to allow a multidisciplinary approach to the management of this clinical condition; the collection of further evidence about efficacy, cost-effectiveness, and safety; and the guarantee of an equal access to clinical pathways and drugs for all the patients.

Notwithstanding, current evidence justifies a positive ethical evaluation of the use of belimumab.

## 4. Discussion

Belimumab represents the first drug approved in the last 50 years specifically to treat patients with active, autoantibody positive SLE who are receiving standard therapy. Prior to belimumab, the last drugs approved by the FDA were Plaquenil (hydroxychloroquine) and corticosteroids in 1955. This delay of development of new lupus drugs could be attributed in conducting phase III studies for regulatory approval. In fact, these studies have an intrinsic complexity related to the particular characteristics of such a complex disease as SLE and to the uneasy definition of the endpoints used to evaluate the efficacy of drugs.

The results of the HTA report presented in this paper show the advantages of belimumab by demonstrating its efficacy, cost-effectiveness, and ethical value which make it a useful therapeutic option with the potential to modify the course of SLE. With respect to efficacy, the HTA report by NICE [49] highlighted that there is an evidence of the clinical effectiveness of belimumab, although a greater consistency of results was observed in BLISS-52 trial, which is not as representative as BLISS-76 of European population. NICE assessment concluded that belimumab was not cost-effective in comparison to SoC but judged appropriately the projection of data about outcomes from short to long term. Notwithstanding, NICE appraisal highlighted different concerns related to the model and its parameters, which may lead to either an over- or an underestimation of the ICER. In particular, since the discontinuation rate could have been underestimated, the ICER has been overestimated. This is relevant also because the discontinuation rate is likely to be higher as shown from the phase II extension study and agreed by clinicians [49].

Finally, the NICE HTA report considered the characteristics of the population affected by the disease and the lack of other licensed treatments. Furthermore, belimumab was considered steroid sparing with the potential to reduce the side effects of these drugs.

Our report thoroughly dealt with epidemiological, economic, organizational, and ethical implications of the use of belimumab in the Italian context, making it possible to support a final positive opinion about the drug.

In fact SLE is a highly health-threatening disease which mainly affects not only women of childbearing age, but also adult males, children, and adolescents. The disease is also burdened with high social and health services costs. Chronic treatment with standard care exposes patients to health risks and patients with high disease activity need alternatives to drug holidays or the increase in the dosage of other drugs. In some cases clinicians use therapies which are not indicated for SLE (off-label drugs), such as rituximab.

Our literature review showed that the prevalence of SLE is about 47 per 100,000 for a total of 28,500 patients in Italy. About 50% of patients are affected by the active form despite SoC. These patients, if positive to anti-dsDNA and with low complement, are eligible to receive belimumab. Furthermore SLE determines an increase in mortality and work disability with costs varying according to disease severity and the development of flares. According to efficacy data, belimumab in association with SoC would prevent 4,742 flares in three years and would be cost-effective. The definition of a clear and efficient treatment pathway for SLE would be worthwhile and requires the involvement of GPs as well as several specialists. Furthermore, belimumab could improve the QoL with positive ethical implications.

In conclusion, no other treatment obtained similar significant or comparable results. However there is a lack in long term efficacy data and also the evidence of the correlation between the SRI and survival should be strengthened. Furthermore safety profile was studied only for a maximum period of seven years of follow-up, which is still inadequate given the chronic nature of the disease. Efficacy data only come from studies versus placebo, since no other treatment proved a significant effect on the control of the disease.

Despite these limits, the strength of this work was the collection, combination, and synthesis of all available data which are important in order to support the sustainable introduction of a new drug. In this view, belimumab may be an innovative and important drug, and postmarketing research will play a key role in updating the HTA and further supporting decision making.

In conclusion this project demonstrates that belimumab may deserve to be introduced in the care of SLE patients in Italy. Our work suggests that tools such as HTA, characterized by a comprehensive approach to the evaluation of health technologies, should be used and implemented in the view of supporting an informed and evidence based decision making process [50].

## 5. Conclusion

The HTA described in this paper shows the value of belimumab and gives important information for its proper use. In particular, the assessment demonstrated that belimumab may prevent flares and is cost-effective in patients with systemic lupus erythematosus who have a highly active disease despite standard of care.

## Figures and Tables

**Table 1:** Model features.

Patients	50,000

Maximum treatment period with belimumab	10 years

Maximum effect related to belimumab	Lifetime

Subgroup	Patient with low complement and anti-dsDNA

Responder rule	Reduction SELENA-SLEDAI ≥ 4 at 24th week

Natural history model	JH-AMS∗ forced in, involvement removed

Long term disease activity model	Adjusted natural history models (NHM)

One-year steroid model	NHM

∗Johns Hopkins University (JH) Adjusted Mean SLEDAI (AMS).

**Table 2:** Main characteristics and results of the randomized controlled trials on belimumab.

Study name (phase)	Study design	Study population	Main results
LBSL02 (Phase II) [41]	Randomized, double blinded, controlled (belimumab 10 mg/kg + SoC versus belimumab 4 mg/kg + SoC versus belimumab 1 mg/kg + SoC versus placebo + SoC)	449 adult subjects with positive antibodies or positive antibodies history and SELENA SLEDAI flare index ≥ 4 from Canada and US	No significant differences in improvement of the SELENA SLEDAI flare index by week 24 and no significant differences in the occurrence of flares by week 52. Evidence of a greater benefit from belimumab in patients currently positive for autoantibodies

C1056/BLISS-76 (Phase III) [42]	Randomized, double blinded, controlled (belimumab 10 mg/kg + SoC versus belimumab 1 mg/kg + SoC versus placebo + SoC)	819 adult subjects with positive antibodies and SELENA SLEDAI flare index ≥ 6 from 19 countries (Europe and North America)	Response at week 52: 43% in the 10 mg/kg belimumab group versus 34% in placebo group, *P* = 0.021. Belimumab reduced the number of severe flares (21% in the 10 mg/kg belimumab group compared to 27% in placebo group, *P* > 0.05)

C1057 BLISS-52 (Phase III) [43]	Randomized, double blinded, controlled (belimumab 10 mg/kg + SoC versus belimumab 1 mg/kg + SoC versus placebo + SoC)	865 adult subjects with positive antibodies and SELENA SLEDAI flare index ≥ 6 from 13 countries (East Europe, Asia, and South America)	Response at week 52: 58% in the 10 mg/kg belimumab group versus 44% in placebo group, *P* < 0.001. Belimumab significantly reduced the number of severe flares (14% in the 10 mg/kg group compared to 23% in placebo group, *P* = 0.006). Belimumab allowed a greater number of patients to reduce the prednisone dosage below 7.5 mg/die (19% in the 10 mg/kg group compared to 12% in placebo group, *P* = 0.053)

**Table 3:** Adverse effects more commonly reported in LBSL02, C1056, and C1057 studies-combined analysis [12].

Symptom	Placebo *N* = 675	Belimumab 1 mg/kg *N* = 673	Belimumab 10 mg/kg *N* = 674
Headache	140 (20.7%)	138 (20.5%)	142 (21.1%)
Upper airways infection	130 (19.3%)	128 (19.0%)	118 (17.5%)
Arthralgia	112 (16.6%)	100 (14.9%)	109 (16.2%)
Nausea	82 (12.1%)	88 (13.1%)	99 (14.7%)
Urinary tract infection	82 (12.1%)	92 (13.7%)	87 (12.9%)
Diarrhea	62 (9.2%)	81 (12.0%)	80 (11.9%)
Asthenia	70 (10.4%)	71 (10.5%)	66 (9.8%)
Pyrexia	52 (7.7%)	52 (7.7%)	65 (9.6%)

**Table 4:** Base case results (NHS perspective).

	SoC	Belimumab	Difference
Life years	18.99	19.76	0.77
QALYs	10.78	11.31	0.538
Costs	€125,234	€142,921	€17,688
ICUR			€32,859
ICER			€22,990

SoC: standard of care; QALY: quality adjusted life year; ICUR: incremental cost-utility ratio; ICER: incremental cost-effectiveness ratio.

**Table 5:** Main parameters used in the base case scenario.

Age (years)—mean	34.7

Gender (% female)	93.9%

Length of disease (years)—mean	6.6

Age at the moment of the diagnosis (years)—mean	28.1

% Afro-Caribbean	7.3%

SLICC (Systemic Lupus International Collaborating Clinics Damage—mean Index)	0.61

SLEDAI—mean	10.9

Daily steroid dose (mg/day)—mean	11.6

The characteristics are determined by pooling the patient-level data from BLISS-52 and BLISS-76 for placebo and belimumab 10 mg/kg.

## Appendix

### Main Parameters Used in the Base Case Scenario

See Table 5.

## References

[1] Rothfield NF, McCarthy EJ (1989). Systemic lupus erythematosus: clinical aspects and treatment. *Arthritis and Allied Conditions: A Textbook of Rheumatology*.

[2] Gladman DD, Urowitz MB, Rahman P, Ibañez D, Tam L (2003). Accrual of organ damage over time in patients with systemic lupus erythematosus. *Journal of Rheumatology*.

[3] Stoll T, Sutcliffe N, Mach J, Klaghofer R, Isenberg DA (2004). Analysis of the relationship between disease activity and damage in patients with systemic lupus erythematosus—a 5-yr prospective study. *Rheumatology*.

[4] Mosca M, Boumpas D, Bruce IN (2012). Treat-to-target in systemic lupus erythematosus: where are we today?. *Clinical and Experimental Rheumatology*.

[5] Bertsias G, Ioannidis JPA, Boletis J (2008). EULAR recommendations for the management of systemic lupus erythematosus. Report of a Task Force of the EULAR Standing Committee for International Clinical Studies Including Therapeutics. *Annals of the Rheumatic Diseases*.

[6] Bertsias GK, Ioannidis JPA, Aringer M (2010). EULAR recommendations for the management of systemic lupus erythematosus with neuropsychiatric manifestations: report of a task force of the EULAR standing committee for clinical affairs. *Annals of the Rheumatic Diseases*.

[7] Hahn BH, McMahon MA, Wilkinson A (2012). American College of Rheumatology guidelines for screening, treatment, and management of lupus nephritis. *Arthritis Care and Research*.

[8] Doria A, Iaccarino L (2013). Terapia del LES. In: L’impiego di belimumab nel Lupus Eritematoso Sistemico: risultati di una valutazione di HTA. *QIJPH*.

[9] Nikpour M, Urowitz MB, Ibañez D, Gladman DD (2009). Frequency and determinants of flare and persistently active disease in systemic lupus erythematosus. *Arthritis Care and Research*.

[10] Zen M, Bassi N, Nalotto L (2012). Disease activity patterns in a monocentric cohort of SLE patients: a seven-year follow-up study. *Clinical and Experimental Rheumatology*.

[11] European Medicines Agency Benlysta. http://www.ema.europa.eu/ema/index.jsp?curl=pages/medicines/human/medicines/002015/human_med_001466.jsp&murl=menus/medicines/medicines.jsp&mid=WC0b01ac058001d125.

[12] Petri MA, Levy RA, Merrill JT (2010). Belimumab, a BLyS-specific inhibitor, reduced disease activity, flares, and prednisone use in patients with seropositive SLE: combined efficacy results from the phase 3 BLISS-52 and -76 studies. *Arthritis & Rheumatism*.

[13] Petri M (2005). Lupus in Baltimore: evidence-based “clinical pearls” from the Hopkins Lupus Cohort. *Lupus*.

[14] Evidence Review Group Report commissioned by the National Health Service Research and Development Programme on behalf of the National Institute for Health and Clinical Evidence Belimumab for the treatment of active autoantibody-positive systemic lupus erythematosus. http://www.hta.ac.uk/project/2527.asp.

[15] http://www.salute.gov.it/portale/temi/p2_6.jsp?lingua=italiano&id=1767&area=programmazioneSanitariaLea&menu=lea.

[16] Fattore G, Torbica A (2008). Cost and reimbursement of cataract surgery in Europe: a cross-country comparison. *Health Economics*.

[17] Pugliatti M, Sobocki P, Beghi E (2008). Cost of disorders of the brain in Italy. *Neurological Sciences*.

[18] Piscitelli P, Iolascon G, Greco M (2011). The occurrence of acute myocardial infarction in Italy: a five-year analysis of hospital discharge records. *Aging—Clinical and Experimental Research*.

[19] Della Rossa A, Neri R, Talarico R (2010). Diagnosis and referral of rheumatoid arthritis by primary care physician: results of a pilot study on the city of Pisa, Italy. *Clinical Rheumatology*.

[20] Turchetti G, Scalone L, Della Casa Alberighi O (2012). The rationale of pharmacoeconomic analysis in rheumatologic indications. *Clinical and Experimental Rheumatology*.

[21] Turchetti G, Yazdany J, Palla I (2012). SLE and the economic perspective: a systematic literature review and points to consider. *Clinical and Experimental Rheumatology*.

[22] Petri M (2005). Review of classification criteria for systemic lupus erythematosus. *Rheumatic Disease Clinics of North America*.

[23] Petri M, Orbai AM, Alarcón GS (2012). Derivation and validation of the systemic lupus international collaborating clinics classification criteria for systemic lupus erythematosus. *Arthritis & Rheumatology*.

[24] Bertoli AM, Vilá LM, Reveille JD, Alarcón GS (2008). Systemic lupus erythaematosus in a multiethnic US cohort (LUMINA) LIII: disease expression and outcome in acute onset lupus. *Annals of the Rheumatic Diseases*.

[25] Ruperto N, Hanrahan LM, Alarcón GS (2011). International consensus for a definition of disease flare in lupus. *Lupus*.

[26] Swaak AJG, van Den Brink HG, Smeenk RJT (1999). Systemic lupus erythematosus: clinical features in patients with a disease duration of over 10 years, first evaluation. *Rheumatology*.

[27] Ehrenstein MR, Isenberg DA, Isenberg DA, Maddison PJ, Woo P (2004). Systemic lupus erythematosus in adults—clinical features and aetiopathogenesis. *Oxford Textbook of Rheumatology*.

[28] Maddison P, Farewell V, Isenberg D (2002). The rate and pattern of organ damage in late onset systemic lupus erythematosus. *Journal of Rheumatology*.

[29] Faurschou M, Starklint H, Halberg P, Jacobsen S (2006). Prognostic factors in lupus nephritis: diagnostic and therapeutic delay increases the risk of terminal renal failure. *Journal of Rheumatology*.

[30] Korbet SM, Lewis EJ, Schwartz MM, Reichlin M, Evans J, Rohde RD (2000). Factors predictive of outcome in severe lupus nephritis. *The American Journal of Kidney Diseases*.

[31] Petri M (2002). Epidemiology of systemic lupus erytematosus. *Best Practice & Research Clinical Rheumatology*.

[32] Rus V, Maury EE, Hochberg MC, Wallace DJ, Hahn BH (2002). The epidemiology of systemic lupus erythematosus. *Dubois’ Lupus Erythematosus*.

[33] Govoni M, Castellino G, Bosi S, Napoli N, Trotta F (2006). Incidence and prevalence of systemic lupus erythematosus in a district of North Italy. *Lupus*.

[34] Benucci M, Del Rosso A, Li Gobbi F, Manfredi M, Cerinic MM, Salvarani C (2005). Systemic lupus erythematosus (SLE) in Italy: an Italian prevalence study based on a two-step strategy in an area of Florence (Scandicci-Le Signe). *Medical Science Monitor*.

[35] Doria A, Amoura Z, Cervera R (2014). Annual direct medical cost of active systemic lupus erythematosus in five European countries. *Annals of the Rheumatic Diseases*.

[36] Davidson JE, Galway N, Egger PJ The prevalence of systemic lupus erythematosus in Europe: a systematic review and meta-analysis.

[37] Bernatsky S, Boivin J-F, Joseph L (2006). Mortality in systemic lupus erythematosus. *Arthritis and Rheumatism*.

[38] Doria A, Iaccarino L, Ghirardello A (2006). Long-term prognosis and causes of death in systemic lupus erythematosus. *The American Journal of Medicine*.

[39] Katz P, Morris A, Trupin L, Yazdany J, Yelin E (2008). Disability in valued life activities among individuals with systemic lupus erythematosus. *Arthritis Care and Research*.

[40] Human Genome Sciences Arthritis Advisory Committee Meeting Briefing Document for the 16 November 2010 Meeting. http://www.fda.gov/downloads/AdvisoryCommittees/CommitteesMeetingMaterials/Drugs/ArthritisDrugsAdvisoryCommittee/UCM23358.

[41] Wallace DJ, Stohl W, Furie RA (2009). A phase II, randomized, double-blind, placebo-controlled, dose-ranging study of belimumab in patients with active systemic lupus erythematosus. *Arthritis Care and Research*.

[42] Furie R, Petri M, Zamani O (2011). A phase III, randomized, placebo-controlled study of belimumab , a monoclonal antibody that inhibits B lymphocyte stimulator, in patients with systemic lupus erythematosus. *Arthritis & Rheumatology*.

[43] Navarra SV, Guzmán RM, Gallacher AE (2011). Efficacy and safety of belimumab in patients with active systemic lupus erythematosus: a randomised, placebo-controlled, phase 3 trial. *The Lancet*.

[44] Thumboo J, Strand V (2007). Health-related quality of life in patients with systemic lupus erythematosus: an update. *Annals of the Academy of Medicine Singapore*.

[45] Yee C-S, McElhone K, Teh L-S, Gordon C (2009). Assessment of disease activity and quality of life in systemic lupus erythematosus—new aspects. *Best Practice & Research Clinical Rheumatology*.

[46] Strand V, Crawford B, Petri M LBSL02 Study Group. Responders defined by SELENA SLEDAI, BILAG and physicians global assessment of disease activity report stabilization and/or improvement in health-related quality of life (HRQOL) following treatment with belimumab.

[47] Kang YM, Lee KW, Ramiterre EB (2010). Belimumab, a BLySspecific inhibitor, significantly improved physical functioning, fatigue, and other health-related quality of life (HRQoL) measures in patients with seropositive. *International Journal of the Rheumatic Diseases*.

[48] Cegedim Strategic Data Survey Lupus Eritematosus Sistemico. Interviste telefoniche con metodologia CATI (integrate da interviste F2F sugli ospedali piu importanti) su 936 Ospedali.

[49] http://guidance.nice.org.uk/index.jsp?action=byID&o=13307.

[50] Ricciardi W, La Torre G (2010). *Health Technology Assessment. Principi, Dimensioni, Strumenti*.

